# Association Study of *MTHFR* C677T Polymorphism With Homocysteine Level and Coronary Heart Disease in Elderly Patients

**DOI:** 10.1155/crp/6246458

**Published:** 2025-03-16

**Authors:** Li Chen, Yi Jiang

**Affiliations:** Ningbo Medical Center, Lihuili Hospital, Ningbo University, Ningbo, Zhejiang 315040, China

**Keywords:** coronary heart disease, elderly patient, gene polymorphism, homocysteine, methylenetetrahydrofolate reductase

## Abstract

**Objective:** To investigate the relationship between methylenetetrahydrofolate reductase (*MTHFR*) C677T gene polymorphism and coronary heart disease (CHD) in the elderly patients living in the coastal area of eastern Zhejiang Province in China.

**Methods:** From September 2021 to May 2022, 163 elderly patients (male ≥ 55 years old, female ≥ 65 years old) admitted to the cardiology department in the Ningbo Lihuili Hospital were collected. Among these patients, 90 patients were diagnosed with CHD (CHD group) and 79 patients did not have CHD (control group). The homocysteine (Hcy) level was measured by the blood biochemical test, and the *MTHFR* genotype was detected by the PCR fluorescence probe method.

**Results:** Compared with the control group, the CHD group showed a significantly higher distribution frequency of TT genotype (*X*^2^ = 5.137, *p* < 0.05) and a lower frequency of CC genotype (*X*^2^ = 6.560, *p* < 0.05), indicating that elderly people with *MTHFR*677 TT genotype are more likely to have CHD. In addition, the Hcy level of TT genotype in the CHD group and the control group were both obviously higher than that of CT genotype and CC genotype (*p* < 0.05). Finally, the univariate and multivariate logistic regression analyses showed that gender, hypertension, diabetes, and *MTHFR*677 TT genotype were independent risk factors for CHD (*p* < 0.05).

**Conclusion:** *MTHFR* C677T mutation is significantly associated with the serum Hcy, and is an important genetic risk for CHD development in the elderly people living in the coastal area of eastern Zhejiang province, China.

## 1. Introduction

Coronary heart disease (CHD) is caused by coronary atherosclerosis, which narrows or blocks the lumen and leads to relative myocardial ischemia and hypoxia [1, 2]. With associated mortality increasing every year, CHD has emerged as one of the main causes of death in elderly cardiovascular patients worldwide [3–5]. Previous studies have revealed that CHD is caused by both genetic and environmental factors [6–8]. Since the traditional environmental factors reflect only a small part of mechanisms related to the development of CHD, genetic factors are considered to play important roles in the incidence of CHD and the prognosis of patients [9]. Genetic polymorphisms alter the activity of enzymes, and are associated with the biological processes. Previous studies have reported that single-nucleotide polymorphisms (SNP) of some genes, such as Notch1, GATA4, NKX2-5, and TBX5, are significantly associated with the risk of CHD [10, 11]. Therefore, clarifying the specific effects of gene polymorphisms will facilitate the disease prevention and treatment of CHD.

Methylenetetrahydrofolate reductase (MTHFR) catalyzes the reduction of 5,10-methylenetetrahydrofolate to 5-methyltetrahydrofolate and is one of the key enzymes in folate metabolism as well as in the synthesis of purine, DNA, and RNA [12]. The *MTHFR* gene has been mapped to chromosome 1p36.3 and is essential for the methyl cycle which converts homocysteine (Hcy) to methionine [13, 14]. The mutation of *MTHFR* gene would reduce the remethylation of Hcy, leading to a high Hcy level in the body [15–17]. Among various SNP in the *MTHFR* gene, the most clinically significant mutation is the missense mutation C677T, which results in the conversion of amino acid alanine to valine at position 226 in the protein [14]. This polymorphism is associated with reduced enzyme activity, increased concentrations of serum Hcy, and decreased concentrations of folic acid [18]. High Hcy levels would induce endothelial dysfunction and injury followed by platelet activation and thrombus formation, which increase the incidence rate of CHD in different populations [19–21].

Previous studies on the relationship of CHD and *MTHFR* gene polymorphisms showed that the frequencies of MTHFR C677T genotype vary markedly across different regions and ethnic groups, and did not pay particular attention to the age of the patient [22–25]. Therefore, accurate information on the distribution of *MTHFR* polymorphism as well as the influence of *MTHFR* polymorphisms on CHD patients with specific ages will be beneficial for gene–disease association studies and health impact assessment.

In this study, we selected the elderly population living in the coastal area of eastern Zhejiang province in China and explored the correlation of serum Hcy level and *MTHFR* C677T polymorphism with CHD susceptibility. It would evaluate the influence of *MTHFR* C677T genetic variation on the risk of CHD in elderly patients and provide a scientific basis for the prevention and treatment of CHD in this region of China.

## 2. Material and Methods

### 2.1. General Information

From September 2021 to June 2022, 169 elderly patients were admitted to the cardiology department in the Ningbo Lihuili Hospital. Among these patients, 90 patients diagnosed with CHD were collected in the CHD group, and 79 patients without CHD were collected in control group. Inclusion criteria: (1) Age: male ≥ 55 years old, female ≥ 65 years old; (2) All patients underwent coronary angiography. Among the right coronary artery, left main artery, left anterior descending branch, circumflex branch, and their main branches, one or more branches with stenosis > 50% were diagnosed as the pathological group, excluding the coronary artery slow flow, X syndrome, etc. The other patients were collected in the control group. Patients with pregnancy, stroke, hepatic or renal failure, severe infection, tumor, and severe heart failure (LVEF < 35%) were excluded. The patients were all unrelated individuals of the Han population living in the eastern Zhejiang province, and were all informed consent to this study. This study protocol was approved by the Ethics Committee at Ningbo Medical Center Lihuili Hospital (KY2022SL328-02).

### 2.2. Biochemical Analysis

The morning fasting venous blood of all patients was collected on the second day after hospitalization, and was used for routine blood biochemical tests, including blood glucose, total cholesterol (TC), triglyceride (TG), low-density lipoprotein cholesterol (LDL-C), high-density lipoprotein cholesterol (HDL-C), apolipoprotein A (ApoA), apolipoprotein B (ApoB), apolipoprotein E (ApoE), lipoprotein a (Lp (a)), uric acid, and blood Hcy.

### 2.3. *MTHFR* C677T Genotype Detection

Genomic DNA was extracted from peripheral blood samples using the TIANamp Blood kit (TIANGEN, Beijing, China). 2 μL genomic DNA was mixed with 23 μL reaction solution of Human *MTHFR* gene detection kit (YZYmed, Wuhan, China) for *MTHFR* genotyping detection. The reaction was performed on the light quantitative PCR instrument following the manufacturer's instructions. Typical PCR amplification condition is 5 min at 95 °C, followed by 40 cycles of 95 °C for 15 s, 60 °C for 60 s. The fluorescence signals of samples were collected through FAM and VIC channels. PAM Ct ≤ 36 with VIC Ct > 36 is classified as *MTHFR*677 CC genotype, PAM Ct ≤ 36 with VIC Ct ≤ 36 is classified as CT genotype, while PAM Ct > 36 with VIC Ct ≤ 36 is classified as TT genotype.

### 2.4. Statistical Analysis

SPSS21.0 statistical software was used to analyze the biochemical data in this study. Continuous variables are presented as mean ± standard deviation (SD), while categorical variables are presented as frequencies and percentiles. If normally distributed, the *T*-test was applied for comparison of continuous variables among two groups. For continuous variables associated with a non-normal distribution, the Mann–Whitney *U* test was used. Between-group differences concerning categorical variables were assessed with a chi-squared test. Multivariate logistic regression analysis was used to analyze the risk factors of CHD. *p* < 0.05 was considered to be statistically significant.

## 3. Results

### 3.1. Comparison of Patient Clinical Data Between CHD and Control Groups

We collected the clinical data from a total of 179 patients. The basic characteristics of the study subjects are shown in Table 1. Statistical analysis revealed a significant difference in the sex ratio in which the majority of CHD patients were males as compared with the control group (*p*=0.023). In addition, the number of patients with hypertension and diabetes in the CHD group was also significantly higher than that in the control group (*p* < 0.05).

### 3.2. Comparison of the Serum Hcy Level Between CHD and Control Groups

In this study, the serum Hcy level in the CHD group is higher than that in the control group. Further data analysis revealed that the Hcy level of the *MTHFR*677 TT genotype was significantly higher than that of the CC genotype and CT genotype in both CHD and control groups (*p* < 0.05) (Table 2).

### 3.3. *MTHFR* C677T Genotype Distribution

In the CHD group, the numbers of patients with *MTHFR*677 CC, CT, and TT genotypes are 23, 43, and 24, respectively. By contrast, there are 35, 34, and 10 patients with CC, CT, and TT genotypes in the control group (Table 3). Statistical analysis showed that there is a significant difference in the frequency of *MTHFR*677 CC and TT genotype between the CHD group and the control group (*p* < 0.05), but no difference in CT genotype between these two groups (*p* > 0.05; Table 3). The frequency of TT genotype in CHD group is significantly higher than that in control group, while the CC genotype frequency in the CHD group is significantly lower compared with the control group (Table 3). Compared with the CC genotype, the TT genotype is an important risk factor for CHD (OR = 3.65, 95% CI: 1.475–9.038, *p*=0.004), while the CT genotype is not (OR = 1.92, 95% CI: 0.963∼3.845, *p*=0.063) (Table 4). Moreover, the proportion of T alleles (the number of T alleles vs. the number of total alleles) in the CHD group is significantly higher than that in the control group (Table 4). These results indicated that the T allele is the predisposing allele for CHD and is probably associated with CHD severity.

### 3.4. Univariate and Multivariate Regression Analysis for Risk Factors of CHD

We carried out univariate and multivariate logistic regression analyses to identify the covariates that independently and significantly contributed to CHD (Table 5). After adjustment of risk factors (age, gender, smoking, hypertension, diabetes, BMI and LDL), both univariate and multivariate logistic regression analyses showed that the MTHFR TT genotype was an independent risk factor for CHD (*p* < 0.01). For other covariates, gender, hypertension, and diabetes were independent risk factors for CHD (all *p* < 0.05), while age, smoking, BMI, LDL, and *MTHFR* CT genotype did no affect CHD (all *p* > 0.05) (Table 5).

## 4. Discussion

CHD is one of the main causes of hospitalization and death in elderly patients. Therefore, it is of great social and economic significance to study its pathogenesis. In our study, we explored the correlation of *MTHFR* C677T polymorphism with CHD susceptibility in the elderly patients living in the coastal area of Zhejiang province in China. To our best knowledge, this study is the first report to analyze the relationship between the *MTHFR* polymorphism and CHD risk in this specific region. Our study revealed that the CHD group showed a significantly higher distribution frequency of TT genotype and a lower frequency of CC genotype comparing the control group. In addition, the Hcy level of TT genotype in the CHD group and the control group were both obviously higher than that of CT genotype and CC genotype. These results indicated that *MTHFR* polymorphism is an important risk factor for CHD, and elderly people with *MTHFR*677 TT genotype are more likely to have CHD.

There are many independent risk factors for CHD, including age, gender, smoking, LDL, familial genetic factors, hypertension, and diabetes [7, 8]. Consistent with previous studies, our results showed that the number of male patients is significantly higher than that of female patients in the CHD group. In addition, patients with hypertension and diabetes in the CHD group are also significantly more than those in the control group. However, age, smoking, and LDL are not risk factors for CHD in our study (all *p* > 0.05) (Table 5). The reason may be the small sample size which includes only 163 patients in this study, leading to instability and uncertainty in data analysis. Therefore, more patient samples are required to obtain more reliable analysis results.

Previous studies showed that high Hcy is an independent risk factor for CHD [16]. The in vivo metabolism of Hcy is mainly through the demethylation and sulfur-transfer pathway which requires active folic acid (5-methyltetrahydrofolate) as the substrate. The N5,10-methylenetetrahydrofolate reductase encoded by the *MTHFR* gene is the key enzyme for the synthesis of active folic acid [13]. The C677T mutation of the *MTHFR* gene reduces the thermostability of the enzyme, which in turn reduces its enzyme activity, resulting in high Hcy [26]. Consistent with the previous studies [16], the serum Hcy level in the CHD group is higher than that in the control group in this study. In-depth data analysis revealed that the Hcy level of the *MTHFR* 677 TT genotype was significantly higher than that of CT heterozygotes and CC genotype in both CHD and control groups, suggesting that *MTHFR* C677T mutation is an important factor for the increase of Hcy level.

Wu et al. reported that the *MTHFR* C677T polymorphism was closely associated with CHD in the population living in Gansu province in China [24]. Ramkaran et al. found that the higher frequency of the *MTHFR* 677 variant allele might be a contributing factor to the higher occurrence of CHD in young South African Indians [27]. In addition, a meta-analysis observed an obvious association between the *MTHFR* C677T polymorphism and CHD risk in the Chinese population [28]. Consistent with these studies, in our study, the proportion of MTHFR677 TT genotype (26.67%) in the CHD group was significantly higher than that in the control group (12.66%), whereas the proportion of CC type (25.56%) in the CHD group was significantly lower than that in the control group (44.3%). Moreover, we noticed that the proportion of T alleles in the CHD group was significantly higher than that in the control group (Table 4). Taken together, our study revealed that *MTHFR* C677T mutation would elevate the serum Hcy level in the body, thus leading to an increase in the incidence rate of CHD.

According to the results of our study, the Hcy level and *MTHFR* C677T polymorphism should be fully considered as important risk factors for the prevention and treatment of CHD. Early prevention of CHD is more necessary for elderly patients with the *MTHFR*677 TT genotype. Restricted by sample size and region, this study still has some limitations. More patient samples from more extensive regions are needed for further research to obtain a definite conclusion.

## Figures and Tables

**Table 1:** Comparison of the demographic data of the CHD and control groups.

Characteristics	Control (*n* = 79)	CHD (*n* = 90)	*t* (*X*^2^)	*p* value
Gender (M/F)	40/39	61/29	5.143	**0.023**
Age (years)	70.19 ± 7.74	69.41 ± 7.28	0.673	0.502
Smoking	16	27	2.107	0.147
Hypertension	49	70	5.011	**0.025**
BMI	25.19 ± 3.09	24.98 ± 2.86	0.448	0.655
Diabetes	13	27	4.272	**0.039**
FBG	5.50 ± 1.41	5.83 ± 2.05	−1.230	0.220
HbA1c	5.91 ± 0.74	6.26 ± 1.31	−2.219	**0.028**
TC (mmol/L)	4.06 ± 1.03	3.91 ± 1.09	0.891	0.374
TG (mmol/L)	1.26 ± 0.72	1.36 ± 0.79	−0.902	0.376
HDL-C (mmol/L)	1.08 ± 0.24	1.08 ± 0.22	0.052	0.958
LDL-C (mmol/L)	2.45 ± 0.71	2.37 ± 0.74	0.758	0.449
ApoA (g/L)	1.16 ± 0.27	1.16 ± 0.23	−0.240	0.810
ApoB (g/L)	0.80 ± 0.20	0.78 ± 0.21	0.532	0.595
ApoE (g/L)	34.59 ± 10.43	35.39 ± 8.10	−0.547	0.586
Lp(a) (g/L)	0.17 ± 0.15	0.20 ± 0.17	−0.855	0.394
UA (mmol/L)	360.92 ± 96.11	379.33 ± 123.30	−1.072	0.285

*Note:* Statistically significant values (*p* < 0.05) are highlighted in bold.

**Table 2:** Comparison of the Hcy level between the CHD and control groups with different *MTHFR* C677T genotype (*X* ± *s*, μmol/L).

Group	Cases	Hcy	Genotype		
			CC	CT	TT
CHD group	90	14.17 ± 9.15^a^	10.92 ± 1.82	11.81 ± 3.88	16.59 ± 14.97^bc^
Control group	79	12.36 ± 8.43	11.28 ± 4.10	11.76 ± 3.29	24.49 ± 9.10^bc^

^a^Comparison with control group, *p* < 0.05.

^b^Comparison with CC genotype, *p* < 0.05.

^c^Comparison with CT genotype, *p* < 0.05.

**Table 3:** Comparison of the genotype distribution between the CHD and control groups.

Group	Cases	CC genotype		CT genotype		TT genotype	
		Cases	Incidence (%)	Cases	Incidence (%)	Cases	Incidence (%)
CHD	90	23	25.56	43	47.78	24	26.67
Control	79	35	44.30	34	43.04	10	12.66
*X* ^2^		6.560		0.381		5.137	
*p*		**0.01**		0.537		**0.023**	

*Note:* Statistically significant values (*p* < 0.05) are highlighted in bold.

**Table 4:** Relationship between *MTHFR* gene polymorphism and CHD.

Genotype	Control group	CHD group	OR value	95% CI	*p* value
CC	35	23	1.00		
CT	34	43	1.92	0.963∼3.845	0.063
TT	10	24	3.65	1.475∼9.038	**0.004**
CT + TT	44	67	2.31	1.210∼4.434	**0.011**
C allele^a^	104	89	1.00		
T allele^b^	54	91	1.96	1.268∼3.057	**0.002**

*Note:* Statistically significant values (*p* < 0.05) are highlighted in bold.

^a^The number of C allele (one allele in CT genotype and two alleles in CC genotype).

^b^The number of T allele (one allele in CT genotype and two alleles in TT genotype).

**Table 5:** Univariate and multivariate regression analyses for risk factors of CHD.

Variable	Univariate			Multivariate		
	OR	95% CI	*p -*value	OR	95% CI	*p -*value
Age	0.986	0.947–1.027	0.499	0.976	0.928–1.028	0.359
Gender	0.488	0.261–0.910	**0.024**	0.436	0.197–0.966	**0.041**
Smoking	1.687	0.830–3.433	0.149	1.269	0.513–3.141	0.606
Hypertension	2.143	1.093–4.202	**0.027**	2.547	1.144–5.674	**0.022**
Diabetes	2.176	1.032–4.589	**0.041**	2.414	1.030–5.661	**0.043**
BMI	0.977	0.882–1.082	0.653	0.940	0.835–1.057	0.302
LDL	0.850	0.558–1.294	0.447	0.949	0.598–1.506	0.824
Genotype CC	1			1		0.004
Genotype CT	1.92	0.963–3.845	0.063	1.521	0.714–3.237	0.277
Genotype TT	3.65	1.475–9.038	**0.004**	5.395	1.995–14.592	**0.001**

*Note:* Statistically significant values (*p* < 0.05) are highlighted in bold.

## Data Availability

The data that support the findings of this study are available from the corresponding author upon reasonable request.

## Nomenclature

CHD: Coronary heart disease
Hcy: Homocysteine
*MTHFR*: Methylenetetrahydrofolate reductase
TC: Total cholesterol
TG: Triglyceride
LDL-C: Low-density lipoprotein cholesterol
HDL-C: High-density lipoprotein cholesterol
ApoA: Apolipoprotein A
ApoB: Apolipoprotein B
ApoE: Apolipoprotein E
Lp (a): Lipoprotein a.
